# Composition and Biological Activities of *Murraya paniculata* (L.) Jack Essential Oil from Nepal

**DOI:** 10.3390/medicines3010007

**Published:** 2016-02-26

**Authors:** Noura S. Dosoky, Prabodh Satyal, Tilak P. Gautam, William N. Setzer

**Affiliations:** 1 Department of Chemistry, University of Alabama in Huntsville, Huntsville, AL 35899, USA; nsd0003@uah.edu (N.S.D.); ps0013@uah.edu (P.S.); 2 Department of Botany, Tribhuvan University, MMAMC, Biratnagar 56600, Nepal; tilakg673@gmail.com

**Keywords:** *Murraya paniculata*, GC-MS, essential oil composition, hierarchical cluster analysis, antifungal, brine shrimp lethality, nematicidal

## Abstract

*Murraya paniculata* (L.) Jack, a small tropical evergreen shrub growing in Nepal, has numerous uses in traditional medicine for treatment of abdominal pain, diarrhea, stomach ache, headache, edema, thrombosis, and blood stasis. The present study investigated the chemical composition and bioactivities of the leaf essential oil from *M. paniculata* from Nepal. The essential oil from leaves was obtained by hydrodistillation and a detailed chemical analysis was conducted by gas chromatography-mass spectrometry (GC-MS). The essential oil was screened for antimicrobial activity using the microbroth dilution test, for nematicidal activity against *Caenorhabditis elegans*, and for lethality against brine shrimp (*Artemia salina*). A total of 76 volatile components were identified from the essential oil. The major components were methyl palmitate (11.1%), isospathulenol (9.4%), (*E*,*E*)-geranyl linalool (5.3%), benzyl benzoate (4.2%), selin-6-en-4-ol (4.0%), β-caryophyllene (4.0%), germacrene B (3.6%), germacrene D (3.4%), and γ-elemene (3.2%). The essential oil showed no antibacterial activity, marginal antifungal activity against *Aspergillus niger* (MIC = 313 μg/mL), a moderate activity against *A. salina* (LC_50_ = 41 μg/mL), and a good nematicidal activity against *C. elegans* (LC_50_ = 37 μg/mL).

## 1. Introduction

The genus *Murraya* (Rutaceae) is made up of about 14 species. *Murraya paniculata* (L.) Jack is a small tropical evergreen shrub, native to the tropical and subtropical parts of the world, including southern China, Taiwan, India, Nepal, Northeastern Pakistan, Sri Lanka, Southeastern Asia (*i.e.*, Cambodia, Laos, Myanmar, Thailand, Vietnam, Indonesia, Malaysia, and the Philippines), and Northern Australia. It is widely naturalized in the southern part of Australia, Southeastern USA and Central America. *M. paniculata* is also known as *Chalcas exotica, Chalcas paniculata*, and *Camunium exoticum* [1]. *M. paniculata* is commonly known as orange jasmine or mock orange. In Nepal, it is known as *bajardante* [2]. The average shrub can grow up to 7 m high. Morphologically, the plant can be distinguished by its alternate, glabrous, and glossy leaves that are once-compound, occurring in 3–7 oddly pinnate leaflets. Leaflets are elliptic to cuneate-obovate, 2–9 cm long × 1.5–6 cm wide [3,4]. *M. paniculata* blooms throughout the year. Inflorescences are terminal, corymbose, few-flowered, and dense. Flowers are pentamerous, bisexual, and sweetly fragrant. Petals are 12–18 mm long, narrowly elliptic to oblanceolate, curved backwards, and white to fading cream in color. The fruit is a fleshy berry, oblong-ovoid, red to orange, and grows up to 2.5 cm in length [3,4].

For many years, *M. paniculata* has been used as an ornamental and a medicinal plant [5]. Due to its hardiness and wide range of soil tolerance, orange jasmine is commonly used as a hedge. The leaves have been used as a food additive in many Indian and Malay dishes due to their strong fragrance [6]. *M. paniculata* is commonly used in traditional medicine for treatment of diarrhea, abdominal pain, stomach ache, dysentery, headache, edema, thrombosis, and stasis of blood. Moreover, it was used as a detoxication agent, anticonvulsant, local anesthetic, and expectorant. Previous reports have shown that the extracts from bark and leaf are stimulant and astringent, and had antinociceptive [7], anti-inflammatory, antidiarrheal [8], antitrypanocidal, antidiabetic, antimalarial, antibacterial, antifungal, and antioxidant activities [9,10]. The essential oil was reported to possess anti-amebic activity [11]. Pangnakorn and Poonpaiboonpipattana reported that the aqueous extract of *M. paniculata* leaves possesses phytotoxic effects on seed germination and seedling growth of *Bidens pilosa*, *Amarathus spinosus*, *Echinochloa crusgalli*, and *Chloris barbata* [12].

*M. paniculata* has been the subject of several phytochemical studies. The leaf extract was reported to contain coumarins [13,14] and flavonoids [15,16,17]. The components of leaf essential oils of *M. paniculata* from Bangladesh [18], China [19], Cuba [20], and Nigeria [5] have been previously reported. However, many factors, including provenance, weather, soil conditions, time of harvest, and the drying technique, can change the chemical composition and yield of essential oils [21]. *M. paniculata* has been described as synonymous with *M. exotica* [22], but this has been controversial and has recently been challenged [23]. The current study was conducted to investigate the composition of the leaf essential oil of *M. paniculata* from Nepal as well as its biological activities.

## 2. Materials and Methods

### 2.1. Plant Material

Leaves of *Murraya paniculata*, collected from city of Biratnagar (26°28′ N, 87°16′ E, and 1072 m above sea level), Morang district, Koshi Zone, Nepal in May 2011, were used in this study. The plant material was identified by Tilak P. Gautam and a voucher specimen has been deposited in the Botany Department, MMAMC Campus, Biratngar, Nepal. The essential oil was obtained from fresh leaf samples (100 g) that were crushed and hydrodistilled using a Clevenger-type apparatus for 4 h. The clear pale-yellow essential oil (1.0 g) produced was stored at 4 °C until analyzed (July 2011).

### 2.2. Gas Chromatographic–Mass Spectral Analysis

The essential oil of *M. paniculata* was analyzed by GC-MS using an Agilent 6890 GC with Agilent 5973 mass selective detector (Agilent Technologies, Santa Clara, CA, USA), an HP-5ms fused silica capillary column and an Agilent ChemStation data system [MSD, operated in the EI mode (electron energy = 70 eV), scan range = 40–400 amu, and scan rate = 3.99 scans/s], and an Agilent ChemStation data system as previously described [24]. The GC column was an HP-5ms fused silica capillary with a (5% phenyl)-polymethylsiloxane stationary phase, film thickness of 0.25 μm, a length of 30 m, and an internal diameter of 0.25 mm. The carrier gas was helium with a column head pressure of 48.7 kPa and a flow rate of 1.0 mL/min. Inlet temperature was 200 °C and interface temperature was 280 °C. The GC oven temperature program was used as follows: 40 °C initial temperature, hold for 10 min; increased at 3 °C/min to 200 °C; increased 2°/min to 220 °C. A 1% *w*/*v* solution of the sample in chloroform was prepared and 1 μL was injected using a 10:1 split ratio. Identification of the oil components was based on their retention indices (RI) and by comparison of their mass spectral fragmentation patterns with those reported in the literature [25].

### 2.3. Antimicrobial Screening

The essential oil of *M. paniculata* was screened for antimicrobial activity against *Bacillus cereus* (ATCC No. 14579), *Aspergillus niger* (ATCC No. 16888), and *Candida albicans* (ATCC No. 10231). The minimum inhibitory concentration (MIC) was determined using the microbroth dilution technique as previously reported [26]. For *B. cereus*, a dilution of the essential oil were prepared in cation-adjusted Mueller Hinton broth (CAMBH) beginning with 50 μL of a 1% *w*/*w* solution of the sample in dimethylsulfoxide (DMSO) plus 50 μL CAMBH. The essential oil solution was serially diluted (1:1) in CAMBH in a 96-well plate. Organisms at a concentration of approximately 1.5 × 10^8^ colony forming units (CFU)/mL were added to each well. Plates were incubated at 37 °C for 24 h; the final minimum inhibitory concentration (MIC) was determined as the lowest concentration without turbidity. Gentamicin was used as a positive antibiotic control. Antifungal activity against *C. albicans* was determined as above using yeast-nitrogen base growth medium with approximately 7.5 × 10^7^ CFU/mL; amphotericin B was the positive control. Antifungal activity against *A. niger* was determined as above using potato dextrose broth inoculated with *A. niger* hyphal culture diluted to a McFarland turbidity of 1.0; amphotericin B was the positive control.

### 2.4. Nematicidal Assay

A nematicidal assay using *Caenorhabditis elegans* was done using a modification of the procedure of Park and co-workers [27]. Briefly, a 1% solution of *M. paniculata* leaf oil in dimethylsulfoxide (DMSO) was used to make dilutions for the sample solutions. The sample solutions were prepared in sterile water beginning with 50 µL of the 1% essential oil solution mixed in 50 µL sterile water. This sample solution was serially diluted (1:1) with sterile water in a 96-well plate. Into each well, 10–30 *C. elegans* (mixtures of juvenile and adult nematodes, male:female:juvenile ~1:1:2) per 50 µL of sample solution were added. Sterile water and serially diluted DMSO were used as controls. The dead and living nematodes were counted after 24 h using a microscope. Dead nematodes were identified by their immobility and straight body, even after transfer to clean water. Mean lethal concentration (LC_50_) values were determined using the method of Reed and Muench [28].

### 2.5. Brine Shrimp Lethality Assay

The brine shrimp (*Artemia salina*) lethality test was done using a modification of the procedure of McLaughlin [29]. *A. salina* eggs were hatched in a sea salt solution (Instant Ocean^®^, Spectrum Brands, Inc. Madison, WI, USA) (38 g/L) with an incandescent light bulb as the heat source. After 48 h, the newly hatched nauplii were counted using a micropipette and transferred to 20 mL vials. A total of nine vials, each containing 10 *A. salina* nauplii in 10 mL of sea salt solution (the same as the hatching solution) were prepared. Of these vials, three were labeled as controls with one vial containing no DMSO, a second vial containing 10 μL of DMSO, and the third vial containing 100 μL DMSO. A second set of three replicate vials contained 10 μL of 1% essential oil solution in DMSO, and the remaining three vials were prepared by adding 100 μL of 1% essential oil solution in DMSO. After 24 h, surviving *A. salina* nauplii were counted in each vial and LC_50_ values were determined using the Reed-Muench method [28].

### 2.6. Hierarchical Cluster Analysis

**Figure 1:**
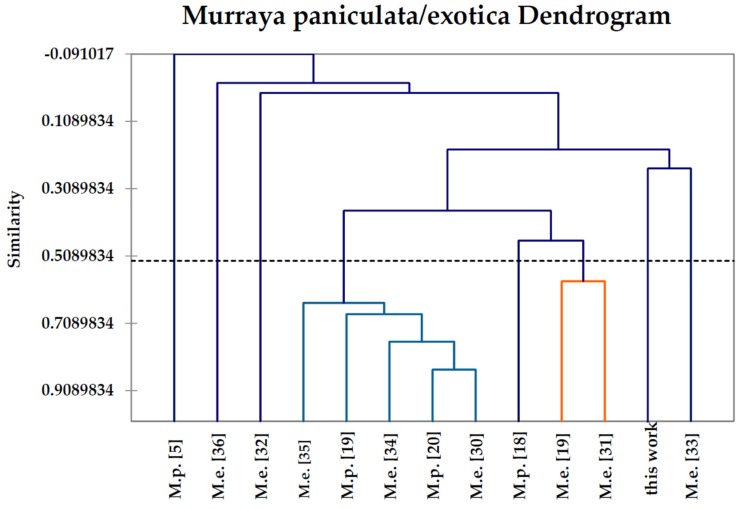
Dendrogram obtained from the agglomerative hierarchical cluster analysis of 13 *Murraya paniculata* leaf essential oil samples.

A total of 12 *M. paniculata* [5,18,19,20] and *M. exotica* [19,30,31,32,33,34,35,36] leaf essential oil compositions from the published literature, as well as the composition from this study, were treated as operational taxonomic units (OTUs). The percentage composition of 35 major essential oil components (α-pinene, methyl salicylate, β-cyclocitral, δ-elemene, α-cubebene, α-copaene, β-cubebene, β-elemene, β-caryophyllene, cedrene, (*E*)-α-bergamotene, β-humulene, (*E*)-β-farnesene, α-humulene, alloaromadendrene, germacrene D, germacrene B, bicyclogermacrene, α-zingiberene, *trans*-β-guaiene, γ-cadinene, cubebol, δ-cadinene, elemol, (*E*)-nerolidol, spathulenol, caryophyllene oxide, viridiflorol, 1,10-di-*epi*-cubenol, 1-*epi*-cubenol, τ-cadinol, β-eudesmol, α-cadinol, benzyl benzoate, and methyl palmitate) was used to determine the chemical relationship between the various *Murraya* essential oil samples by agglomerative hierarchical cluster (AHC) analysis using the XLSTAT software, version 2015.4.01 (Addinsoft SARL, Paris, France). Pearson correlation was selected as a measure of similarity, and the unweighted pair-group method with arithmetic average (UPGMA) was used for cluster definition. The resulting dendrogram is shown in Figure 1.

## 3. Results and Discussion

The *M. paniculata* leaf essential oil composition is shown in Table 1. The leaf oil was mainly composed of methyl palmitate (11.05%), isospathulenol (9.44%), (*E*,*E*)-geranyl linalool (5.29%), benzyl benzoate (4.20%), selin-6-en-4-ol (4.01%), β-caryophyllene (3.97%), germacrene B (3.62%), germacrene D (3.39%), and γ-elemene (3.19%) ,with other minor constituents (<3%). The current study revealed that the essential oil composition and percentages are significantly different from the previously published reports from Bangladesh and China. The major constituents of the leaf oil of *M. paniculata* of Bangladeshi origin were caryophyllene oxide (16.6%), β-caryophyllene (11.8%), spathulenol (10.2%), β-elemene (8.9%), germacrene D (6.9%), and methylene-6-4-(1-propenylidene)cyclooctene (6.4%) [18], while the main components of Chinese *M. paniculata* essential oil were β-caryophyllene (23.3%), spathulenol (16.1%), (*E*)-α-bergamotene (9.3%), (*E*)-nerolidol (4.6%), and δ-elemene (3.3%) [19]. The leaf oil of *M. paniculata* from Cuba was also rich in β-caryophyllene (29.8%) and spathulenol (5.1%), but had significant quantities of caryophyllene oxide (6.3%), viridiflorol (5.7%), δ-cadinene (5.6%), bicyclogermacrene (5.6%), α-humulene (5.3%), and β-cubebene (5.3%) [20]. The leaf oil of Nigerian *M. paniculata* was mainly composed of β-cyclocitral (22.9%), methyl salicylate (22.4%), (*E*)-nerolidol (11.7%), α-cubebene (7.9%), cubenol (6.8%), β-cubebene (5.8%) and isogermacrene (5.7%) [5].

Since *M. paniculata* has often been classified as a species, if it is synonymous with *M. exotica* [5], it is unclear about which essential oil composition may belong to which, or if they are, indeed, separate species. Lv and co-workers [19] have treated *M. paniculata* and *M. exotica* as separate species and have examined the essential oil compositions of both. These workers found *M. exotica* from Guangxi Province, China, to be qualitatively similar to *M. paniculata* (see above), and was dominated by spathulenol (25.6%), *trans*-β-guaiene (13.7%), β-caryophyllene (11.7%), and bicyclogermacrene (4.1%) [19]. There is much variation in the compositions of *M. exotica* essential oils, however. *M. exotica* oil from Hainan, China, was rich in β-caryophyllene (45.5%) and cedrene (15.1%) [30], while a sample from Guangdong, China, had spathulenol (17.7%), α-pinene (13.2%), caryophyllene oxide (8.6%), and bicyclogermacrene (7.1%) as major components [31]. In order to attempt to sort out the volatile phytochemistry of *Murraya paniculata/exotica*, a hierarchical cluster analysis was carried out on the essential oil compositions of *M. paniculata* and *M. exotica* reported in the literature (Figure 1) [5,18,19,20,30,31,32,33,34,35,36]. The components used in the cluster analysis are summarized in Table 2 and illustrate the chemical differences between these essential oil samples. Although there are only 13 essential oil samples, too few to provide a comprehensive chemotaxonomic representation of this species, this analysis does serve to place *M. paniculata* leaf oil from Nepal into context with previously-reported essential oils of *M. paniculata* and *M. exotica*.

The cluster analysis reveals at least eight chemotypes for the *Murraya paniculata/exotica* complex based on volatiles: (1) a methyl salicylate/β-cyclocitral chemotype represented by the *M. paniculata* sample from Nigeria [5]; (2) a β-humulene chemotype represented by the *M. exotica* sample from India [36]; (3) a chemotype dominated by α-pinene represented by the *M. exotica* sample from Egypt [32]; (4) a cluster rich in β-caryophyllene with *M. paniculata* samples from China [19] and Cuba [20] and *M. exotica* samples from China [30,35] and Cuba [34]; (**5**) a caryophyllene oxide/β-caryophyllene/spathulenol chemotype represented by the *M. paniculata* sample from Bangladesh [18]; (6) a spathulenol-rich cluster with *M. exotica* samples from China [19,31]; (7) a β-caryophyllene/α-zingiberene chemotype represented by the *M. exotica* sample from India [33]; and (8) the sample from Nepal (this work), rich in methyl palmitate. In addition to genetic variation [23], age [37], vegetative cycle stage [38], climate [39], season [40], soil composition [41], and edaphic factors [42] are among several factors responsible for the considerable variation in essential oil compositions [43,44]. Based on the observed composition, the Nepalese *M. paniculata* leaf oil is chemically distinct from previously-reported analyses and may represent a distinct chemotype.

The essential oil of *M. paniculata* was screened for potential antimicrobial activity. Based on our experience [26,45,46], we consider samples to have good antimicrobial activity with MIC < 156 μg/mL, moderate activity with MIC between 156 and 313 μg/mL, and weak activity between 313 and 625 μg/mL. Samples with MIC > 625 we consider to be inactive. Based on these criteria, *M. paniculata* essential oil was inactive against *Bacillus cereus* and *Candida albicans* (MIC = 2500 μg/mL) and marginally antifungal against *Aspergillus niger* (MIC = 313 μg/mL). Nevertheless, the antifungal activity of *M. paniculata* leaf oil was better than many of the essential oils we have tested [47,48], comparable to *Mitracarpus scaber* leaf essential oil (MIC = 313 μg/mL) [49] and *Betula nigra* buds essential oil (MIC = 313 μg/mL) [50], but not as effective as *Pinus roxburghii* cone essential oil (MIC = 39 μg/mL) [51], *Cinnamomum camphora* leaf essential oil (MIC = 19.5 μg/mL) [52], *Curcuma longa* leaf essential oil (MIC = 19.5 μg/mL) [53], or *Canthium subcordatum* fruit essential oil (MIC = 39 μg/mL) [54]. Although in low concentrations, α-humulene (1.1%) and germacrene D (3.4%) may contribute to the antifungal activity of *M. paniculata* leaf oil, both have shown activity against *A. niger* (MIC = 78 and 39 μg/mL, respectively, for α-humulene and germacrene D [47]). Methyl palmitate (11.1% in *M. paniculata* oil) has shown antifungal activity (MIC = 333 μg/mL) against *Blumeria graminis* [55].

*M. paniculata* oil showed moderate activity in the brine shrimp (*Artemia salina*) lethality test with LC_50_ value of 41 μg/mL. Essential oils showing *A. salina* toxicity with LC_50_ < 10 μg/mL are considered very active [56], 10 μg/mL < LC_50_ < 50 μg/mL are moderately active [51,57], and 50 μg/mL < LC_50_ < 100 μg/mL, weakly active. In our nematicidal activity screening against *C. elegans*, we have found LC_50_ values ranging from 18 to 1100 μg/mL (unpublished), and we consider nematicidal activities LC_50_ < 100 μg/mL to be very active, LC_50_ values between 100 and 200 μg/mL to be moderately active [52,58], between 200 and 300 μg/mL to be weakly active, and > 300 μg/mL to be inactive. Thus, *M. paniculata* oil was highly nematicidal to *Caenorhabditis elegans* (LC_50_ = 37 μg/mL). It is difficult to speculate as to which compound(s) in the essential oil may be responsible for the brine shrimp lethality or nematicidal activity; there are many components in the leaf oil and none are especially dominant.

## 4. Conclusion

The leaf essential oil of *Murraya paniculata* growing in Nepal has been analyzed by GC-MS and revealed this to be a distinct chemotype, rich in methyl palmitate. Biological screening of the leaf oil showed it to have good nematicidal activity, marginal activity against brine shrimp and *Aspergillus niger*, and inactive against bacteria. The particular chemotype of this plant could have important implication on its biological activity and traditional medicinal uses.

## Figures and Tables

**Table 1:** Chemical composition of the leaf essential oil of *Murraya paniculata* from Nepal.

RI ^a^	Compound	%
1100	Linalool	1.20
1112	Phenylethyl alcohol	0.73
1138	Benzeneacetonitrile	0.14
1189	α-Terpineol	0.16
1254	Geraniol	0.19
1290	Indole	1.25
1325	*p*-Vinylguaiacol	0.69
1332	Bicycloelemene	0.12
1334	δ-Elemene	3.19
1336	Methyl anthranilate	2.05
1391	β-Elemene	0.38
1398	(*Z*)-Jasmone	0.59
1418	β-Caryophyllene	3.97
1451	*cis*-Murrola-3,5-diene	0.25
1453	α-Humulene	1.10
1476	γ-Gurjunene	1.07
1480	Germacrene D	3.39
1483	α-Curcumene	0.84
1485	β-Selinene	0.16
1491	δ-Selinene	0.52
1496	α-Zingiberene	2.16
1504	Germacrene A	0.42
1509	(*E*,*E*)-α-Farnesene	2.79
1514	Cubebol	0.39
1521	α-Chamigrene	0.91
1523	β-Sesquiphellandrene	0.71
1549	α-Elemol	0.45
1556	Germacrene B	3.62
1564	(*E*)-Nerolidol	0.43
1571	(3*Z*)-Hexenyl benzoate	0.27
1581	*trans*-Sesquisabinene hydrate	0.16
1583	Caryophyllene oxide	0.40
1586	Phenylethyl tiglate	0.27
1593	Spathulenol isomer	1.39
1606	β-Nootkatol	0.35
1614	Zingiberenol	0.39
1620	Selin-6-en-4-ol	4.01
1624	1,10-di-*epi*-Cubenol	1.09
1628	Isospathulenol	9.44
1639	τ-Cadinol	2.02
1644	*epi*-β-Muurolol	0.34
1647	β-Eudesmol	0.17
1649	τ-Muurolol	1.01
1651	α-Cadinol	1.07
1663	Intermedeol	0.22
1680	Germacra-4(15),5,10(14)-trien-1α-ol	0.12
1689	(2*Z*,6*Z*)-Farnesol	0.22
1713	Eudesma-4,11-dien-2-ol	0.42
1716	(2*E*,6*Z*)-Farnesol	0.26
1720	Nuciferol	2.13
1733	Oplopanone	0.20
1745	Curcumen-12-ol	2.33
1761	Benzyl benzoate	4.20
1771	Isospathulenol isomer	0.93
1852	Phenylethyl octanoate	2.39
1865	Benzyl salicylate	0.29
1919	Methyl palmitate	11.05
1953	Phenylethyl salicylate	0.13
1957	Palmitic acid	0.80
1996	Ethyl palmitate	0.39
2031	(*E*,*E*)-Geranyl linalool	5.29
2094	Methyl linoleate	0.73
2100	Methyl linolenate	1.30
2110	Phytol	2.11
2123	Methyl stearate	0.60
2130	Osthole	2.58
2232	Isogeigerin	0.24
2238	Suberosin epoxide	0.82
2268	7-Methoxy-6-(3′-metylbuta-1′,3′-dienyl)coumarin	0.47
2270	Muurialongin	0.59
2407	Minimicrolin isovalerate	0.45
2416	Paniculol	tr ^b^
2504	Octyl palmitate	0.59
2705	Octyl stearate	0.21
2711	Murpaniculol senecioate	tr
2828	Squalene	0.25
Compounds Identified		76 (91.5%)

^**a**^ RI determined with respect to a homologous series of *n*-alkanes on an HP-5ms column.^**b**^ tr = “trace” (<0.05%).

**Table 2:** Components used in the hierarchical cluster analysis of *Murraya paniculata*/*Murraya exotica* leaf essential oils.

**Compound**	**M.p. ^a^**	**M.p.**	**M.p.**	**M.p.**	**M.p.**	**M.e. ^b^**	**M.e.**	**M.e.**	**M.e.**	**M.e.**	**M.e.**	**M.e.**	**M.e.**
	**This**	[18]	[20]	[5]	[19]	[19]	[33]	[34]	[35]	[32]	[31]	[30]	[36]
α-pinene	0	0	0	tr	0	0	0	tr	0	62.5	13.2	0.3	0
Methyl salicylate	0	0	0	22.4	0	0	0	0	0	0	0	0	0
β-Cyclocitral	0	0	0	22.9	0	0	0	0	0	0	0	0	0
δ-Elemene	3.2	3.6	0.4	0	3.3	3.4	5.1	0.2	0	0.4	0	0	0
α-Cubebene	0	3.0	2.2	7.9	0.9	0.1	0	1.0	6.9	0	0	0.4	0
α-Copaene	0	2.3	3.8	0.2	0	0.1	0.5	4.4	1.4	0.4	1.7	0.2	0
β-Cubebene	0	0	5.3	5.8	1.6	0	1.6	10.5	0	1.6	0	0	0
β-Elemene	0.4	8.9	0	0	0.1	2.0	0	0	0	0	0.9	0.1	7.6
β-Caryophyllene	4.0	11.8	29.8	0	23.3	11.7	9.7	24.1	20.3	5.2	4.5	45.5	7.1
Cedrene	0	0	0	0	0	0	0	0	0	0	0	15.1	0
α-(*E*)-Bergamotene	0	0	0	0	9.3	0	2	0	0	0	0	0	0
β-Humulene	0	0	0	0	0	0	0	0	0	0	0	0	40.6
(*E*)-β-Farnesene	0	0	0	0	2.6	0	1.5	0	2.4	0	0	0	0
α-Humulene	1.1	3.1	5.3	tr	0	3.0	0.6	5.8	0	0.8	7.3	0.3	t
Alloaromadendrene	0	0	0	0	0.1	1.9	0	0	5.9	0	0.2	0	0
GermacreneD	3.4	7.0	4.2	0	0.5	2.4	2.6	11.9	0	2.1	0.8	0	0
GermacreneB	0	0	0	0	0	0	3.5	0	0	1.9	0	0	0.9
Bicyclogermacrene	0	0	5.6	0	1.9	4.1	0	11.8	9.6	0	7.1	0	0
α-Zingiberene	2.2	0	0	0	0	0	10	0	12.7	0	0	0	0
*trans*-β-Guaiene	0	0	0	0	0	13.7	0	0	0	0	0	0	0
γ-Cadinene	0	0	2.2	0	0	0	0	tr	2.1	1.1	0	0	0
Cubebol	0.4	0	0	6.8	0	0	0	3.1	0	0	0	0	0
δ-Cadinene	0	0	5.6	tr	2.2	1.2	0	4.4	8.0	0.5	4.4	0	0
α-Elemol	0.5	0	0	0	0.2	1.7	0.2	1.3	0	0	0	0	0.1
(*E*)-Nerolidol	0.4	0	0	11.7	4.6	0.4	27.8	1.5	2.7	0	0	0	0
Spathulenol	0	10.2	5.1	3.6	16.1	25.6	0.9	1.0	6.3	0.5	17.7	4.4	0.1
Caryophyllene oxide	0.4	16.6	6.3	tr	2.8	tr	0.1	0.8	4.0	0.5	8.6	1.3	tr
Viridiflorol	0	2.2	5.7	0	0	0	0.6	0.4	0	0	0	0	0
1,10-di-*epi*-Cubenol	1.1	2.4	1.9	0	0	0.2	0	0	0	0	0	0	0
1-*epi*-Cubenol	0	0	2.5	1.2	0.4	0.1	0	1.1	0	0	0	0	0
τ-Cadinol	2.0	0	4.3	3.2	0	0	2.2	0	0	0.6	3.6	0	0
β-Eudesmol	0.2	0	0	0	2.9	2.2	0	0	0	0	0	0	0
α-Cadinol	1.1	0	1.7	0	0	0	0.2	1.4	0.6	0.3	0	0	0
Benzyl benzoate	4.2	0	0	0	0	0	0	0	0	0	0	0	24.0
Methyl palmitate	11.1	0	0	0	tr	tr	0	0	0	0	0	0	0

^a^ M.p. = Murraya paniculata; ^b^ M.e. = Murraya exotica.

## Abbreviations

The following abbreviations are used in this manuscript:

GC-MS: Gas chromatography–mass spectrometry
RI: Retention indices
ATCC: American type culture collection
DMSO: Dimethylsulfoxide
LC_50_: Median lethal concentration
